# Solution nuclear magnetic resonance spectroscopy on a nanostructured diamond chip

**DOI:** 10.1038/s41467-017-00266-4

**Published:** 2017-08-04

**Authors:** P. Kehayias, A. Jarmola, N. Mosavian, I. Fescenko, F. M. Benito, A. Laraoui, J. Smits, L. Bougas, D. Budker, A. Neumann, S. R. J. Brueck, V. M. Acosta

**Affiliations:** 1 Department of Physics, Harvard University, Cambridge, 02138 MA USA; 2 Center for High Technology Materials, Department of Physics and Astronomy, University of New Mexico, Albuquerque, 87106 NM USA; 3 ODMR Technologies Inc., El Cerrito, 94530 CA USA; 4 Department of Physics, University of California-Berkeley, Berkeley, 94720 CA USA; 5 Johannes Gutenberg Universität Mainz, 55128 Mainz, Germany; 6 Helmholtz Institut Mainz, 55099 Mainz, Germany

**Keywords:** Optical properties of diamond, Solution-state NMR

## Abstract

Sensors using nitrogen-vacancy centers in diamond are a promising tool for small-volume nuclear magnetic resonance (NMR) spectroscopy, but the limited sensitivity remains a challenge. Here we show nearly two orders of magnitude improvement in concentration sensitivity over previous nitrogen-vacancy and picoliter NMR studies. We demonstrate NMR spectroscopy of picoliter-volume solutions using a nanostructured diamond chip with dense, high-aspect-ratio nanogratings, enhancing the surface area by 15 times. The nanograting sidewalls are doped with nitrogen-vacancies located a few nanometers from the diamond surface to detect the NMR spectrum of roughly 1 pl of fluid lying within adjacent nanograting grooves. We perform ^1^H and ^19^F nuclear magnetic resonance spectroscopy at room temperature in magnetic fields below 50 mT. Using a solution of CsF in glycerol, we determine that 4 ± 2 × 10^12 19^F spins in a 1 pl volume can be detected with a signal-to-noise ratio of 3 in 1 s of integration.

## Abstract (web summary)

Nitrogen vacancy (NV) centres in diamond can be used for NMR spectroscopy, but increased sensitivity is needed to avoid long measurement times. Kehayias et al. present a nanostructured diamond grating with a high density of NV centres, enabling NMR spectroscopy of picoliter-volume solutions.

## Introduction

Nuclear magnetic resonance (NMR) spectroscopy is an invaluable analytical tool for determining the composition, structure, and function of complex molecules. However, conventional high-field NMR spectrometers have drawbacks. Owing to their reliance on cryogenic magnets, the best NMR spectrometers are massive, immobile, and expensive. Often they require relatively large quantities of analyte and large (≳10 μl) sample volumes to overcome fundamental sensitivity constraints from inductive detection and low thermal polarization. This limits NMR use in sample-limited analysis and high-throughput screening, where a parallel microfluidic platform would be preferable^1–3^. Microcoil NMR^4–8^ can lower the required analyte volume to ≳1 nl, but still requires a large magnetic field for sensitive spectroscopy. Nuclear hyperpolarization methods and alternative magnetometry technologies^9, 10^ enable certain experiments at lower fields, but there is still no platform that combines the high sensitivity and sub-nl analyte volume necessary for operation in microfluidic assays.

Recently, a new technique has emerged for NMR spectroscopy at the nanometer scale based on optical detection of the electron spin resonances of negatively charged nitrogen-vacancy (NV) color centers in diamond^11–18^. The technique relies on statistical nuclear polarization, which is substantially larger than thermal polarization for nanoscale sensing volumes^19^. Combined with non-inductive optical detection, this renders the NV NMR sensitivity independent of temperature and magnetic field. Previous lines of work used single NV centers or an NV ensemble^11–17^ to detect NMR of nuclei in liquids and thin films across a flat sample–sensor interface. While this was a significant scientific breakthrough, the long measurement time (hours to days) is a liability for many applications.

In this work we demonstrate picoliter (pl) solution NMR using a nanostructured diamond chip. The NMR detection sensitivity depends on the number of NV centers that are located close enough to the diamond surface to sense external spins. To increase this number, the diamond surface is lithographically structured with dense, high-aspect-ratio nanogratings to enhance the sensor–analyte contact area by ≳15×. The nanostructure sidewalls are then doped with a high density of NV centers. The result is tens of millions of NV centers located close enough to the diamond surface (5–20 nm) to detect the NMR spectrum from ~1 pl of fluid lying within the adjacent nanograting grooves. This leads to a corresponding boost in fluorescence signal and reduction in acquisition time. With further improvements in spectral resolution, this platform could enable a wide variety of applications in biochemistry, including pharmacodynamic studies of metabolites and natural products and high-throughput screening for drug discovery.

## Results

### Statistical polarization and thermal polarization

NMR is a powerful analytical technique for non-destructive molecular structure elucidation, but its detection sensitivity is orders of magnitude worse than other analytical chemistry techniques such as mass spectrometry or fluorescence labeling methods^5^. The sensitivity is limited by the small nuclear magnetization. At the highest magnetic field available, *B*_0_ = 24 T, the room-temperature ^1^H thermal polarization is just 10^−4 20^. The sensitivity is further limited by the use of inductive detection, which leads to a frequency-dependent signal-to-noise ratio. One potential remedy is to increase the magnetic field, but despite steady improvements in magnet technology, the signal strength has improved by less than a factor of two over the last 20 years^21^.

**Fig. 1:**
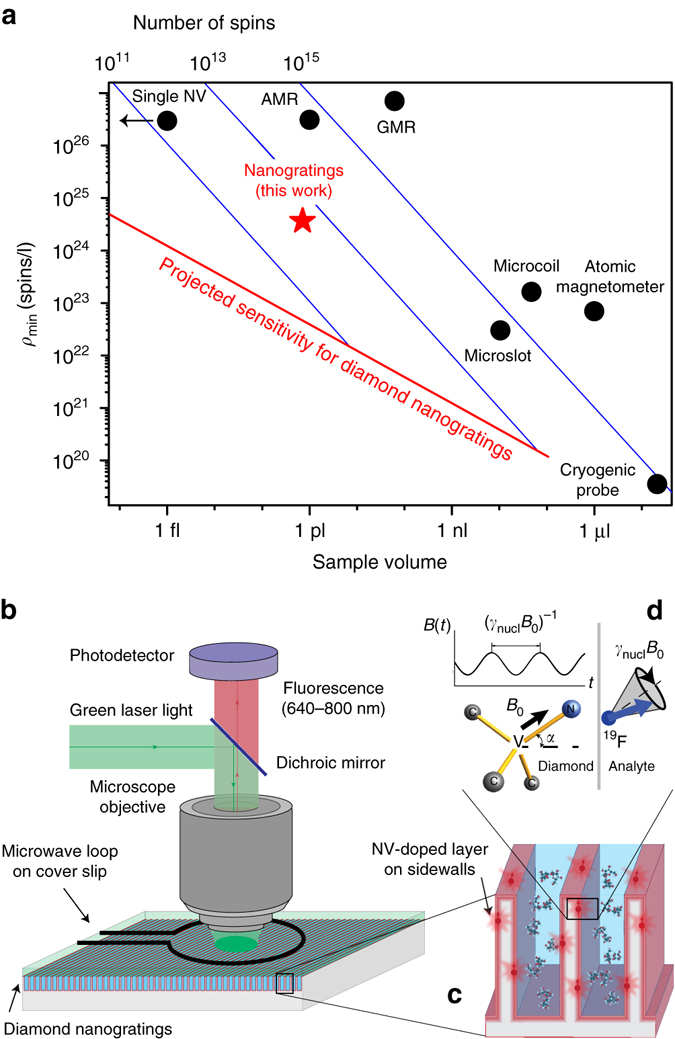
Picoliter NMR. **a** Overview of ambient-temperature NMR techniques for small volumes. *Points* represent experimental values for minimum-detectable nuclear spin concentration in 1 s with SNR = 3 for different techniques: microslot^7^, microcoil^8^, cryogenic probes^22^, atomic vapor magnetometers^25^, giant magnetoresistance (GMR) sensors^23^, anistropic magnetoresistance (AMR) sensors^24^, single NV centers^14^, and NV-doped nanogratings (this work). The *solid red line* is the projected sensitivity for diamond nanogratings (Eq. (1)), exhibiting volume^−1/2^ scaling (see Supplementary Note 1). *Solid blue lines* indicate constant numbers of spins. **b** Epifluorescence diamond NMR set-up. **c** The sensor region consists of dense, high aspect-ratio diamond nanogratings fabricated via interferometric lithography and doped with NV centers. **d** Experimental geometry. The analyte’s precessing nuclear statistical polarization produces an oscillating magnetic field, which is sensed by adjacent near-surface NV centers

Alternative NMR techniques seek to increase nuclear polarization and/or improve signal-to-noise without relying on increasing *B*_0_. The latter can be accomplished by cryogenically cooling the inductive probe^22^ or switching to non-inductive detection modalities, including giant magnetoresistance sensors^23^, anistropic magnetoresistance sensors^24^, or atomic magnetometers^25^. The present NV-based NMR approach uses non-inductive detection to sense the statistical nuclear polarization, an effect that arises from imperfect cancellation of the net magnetization from an ensemble of randomly oriented spins^11, 12^. Statistical polarization is larger than thermal polarization for sufficiently small numbers of nuclear spins (see Supplementary Note 1) and makes the NV NMR sensitivity independent of sample temperature and *B*_0_. Figure 1a summarizes existing NMR techniques for small sample volumes. The NMR sensitivity is characterized by the minimum spin concentration detectable in 1 s at room temperature with signal-to-noise ratio (SNR) of 3.

For NV NMR the minimum detectable spin concentration *ρ*_min_ for SNR = 3 in 1 s is (see Supplementary Note 1):

1 $${\rho _{{\rm{min}}}} = \frac{3}{{P\left( \alpha \right){{\left( {{\mu _0}\hbar {\gamma _{_{{\rm{NV}}}}}{\gamma _{{\rm{nucl}}}}} \right)}^2}}} \times \frac{{d_{_{{\rm{NV}}}}^3}}{{T_{{\rm{tot}}}^2C\sqrt {\eta {N_{_{{\rm{NV}}}}}{N_{\rm{r}}}} }},$$

where $P(\alpha ) = \left\{ {\pi \left[ {8 - 3{{\left( {{\rm{sin}}\,\alpha } \right)}^4}} \right]} \right\}{\rm{/}}128$ is a geometric factor that comes from the angle *α* the N–V axis makes with the diamond surface normal (Fig. 1c, d), *μ*_0_ = 4*π* × 10^−7^ m T/A is the vacuum permeability, *ħ* = 1.055 × 10^−34^ J·s is the reduced Planck constant, *γ*_NV_ = 28.03 GHz/T is the NV gyromagnetic ratio, *γ*_nucl_ is the nuclear gyromagnetic ratio (42.58 MHz/T for ^1^H and 40.08 MHz/T for ^19^F), *d*_NV_ is the characteristic NV depth below the diamond surface, *T*_tot_ is the NV phase accumulation time during a single XY8-N pulse sequence (Fig. 3a), *C* is the NV fluorescence-detected spin contrast, *N*_NV_ is the number of near-surface NV centers in the sensing area, *η* is the mean number of photons collected per NV per readout *(η* < 1), and *N*_r_ is the number of readouts per second.

**Fig. 2:**
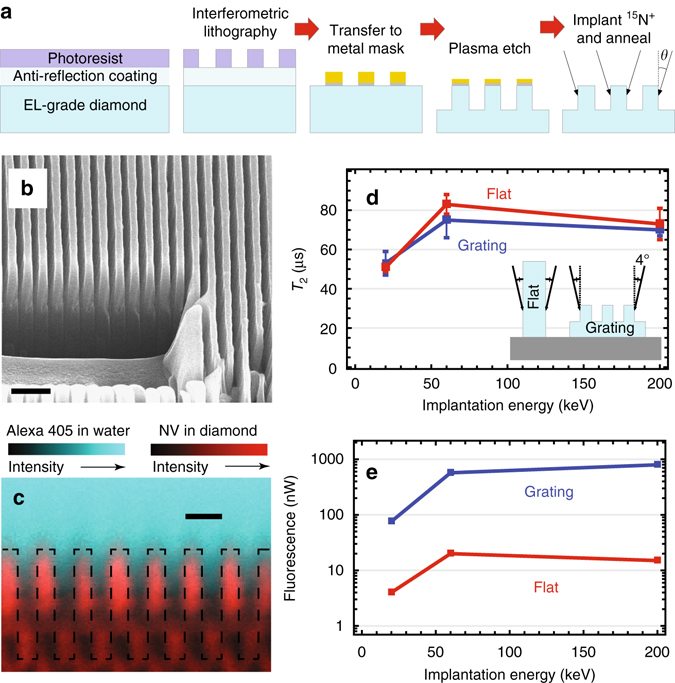
Diamond nanogratings. **a** *Schematic* of large-area nanofabrication process. **b** Scanning electron micrograph of 400 nm pitch diamond nanogratings. Focused ion beam etching prior to imaging enabled visualization of the nanogratings’ cross-section. Scale bar is 1 μm. **c** Confocal microscopy images reveal that fluorescence from dye-stained water originates from areas inside the nanograting grooves, confirming wetting. *Dashed lines* represent the estimated diamond–water boundary. Scale bar is 500 nm. **d** Comparison of *T*_2_, measured with the XY8-22 protocol, and **e** fluorescence intensity between flat and nanograting chips implanted at similar conditions. *T*_2_ can surpass ~100 μs with sufficient decoupling *π*-pulses (see Supplementary Note 2). *Error bars* in **d** represent standard error of the mean

Since ${\rho _{{\rm{min}}}} \propto 1{\rm{/}}\sqrt {\eta {N_{_{{\rm{NV}}}}}}$, the sensitivity can be improved by increasing the sensor surface area, boosting *N*_NV_ for a constant laser spot size. To this end, we nanostructured the diamond surface with high-aspect-ratio nanogratings and doped the sidewalls with a high density of NV centers.

### Diamond chip fabrication and characterization

Nanogratings were etched into the surface of electronic-grade [100]-polished diamond chips using optical interferometric lithography^26, 27^ and diamond plasma etching (see Supplementary Note 2 and refs. ^28, 29^), as shown in Fig. 2a. The nanogratings have a 400 nm pitch and a depth up to 3 μm (Fig. 2b). By varying the resist, postbake, and development conditions, duty cycles of 20–80% are achievable. The nanograting sidewalls were doped with NV centers by implanting ^15^N^+^ ions at angles *θ* = ±4° relative to the substrate surface normal. The angles were chosen to ensure that an entire 3-μm-tall sidewall would be doped. Each chip was implanted with either 20, 60, or 200 keV ion energy, corresponding to ~5, 10, and 20 nm simulated NV depths, respectively (see Supplementary Note 2 for doses and other information). After implantation, the chips were annealed in vacuum at 800–1100 °C to form NV centers^30^. We investigated the analyte/nanograting adhesion (wetting) with confocal microscopy. Water stained with Alexa 405 dye was dispersed on top of an NV-doped nanograting chip. Afterwards, fluorescence from the NV centers (650–800 nm) and dye-stained water (425–500 nm) was simultaneously imaged. We confirmed that the nanogratings were wetting by observing Alexa 405 fluorescence from areas inside the nanograting grooves (Fig. 2c).

**Fig. 3:**
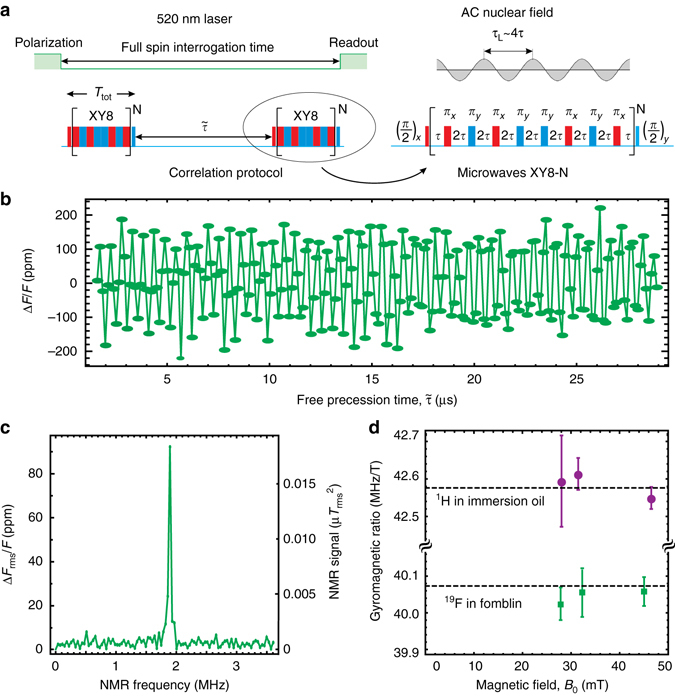
Nanoscale NMR. **a** Sensing protocols: optical pulses are used to pump and probe NV spin state via the spin-dependent fluorescence; microwave multipulse sequences are applied between optical pump and probe pulses. *Red* and *blue color* indicates different microwave phases, which are shifted relative to each other by 90°. NV centers are resonantly tuned to detect a particular nuclear species by setting 4*τ* = *τ*_L_, where 2*τ* is the separation between *π*-pulses and *τ*_L_ is the nuclear precession period. In order to reject common-mode noise the sequences are repeated with the phase of the last *π*/2-pulse shifted by 180°. The resulting signals are then subtracted and normalized to give the measurement results. **b** Time-domain NMR signal for ^19^F nuclei in Fomblin^®^ oil taken using XY8-13 correlation sequence. **c** ^19^F frequency-domain NMR signal at *B*_0_ = 47.1 mT obtained by Fourier transform of the data in **b**. **d** Measured ^1^H and ^19^F gyromagnetic ratios at different *B*_0_ values. *Dashed lines* are literature values^51^. *Error bars* represent standard error of the mean

Optical NMR detection was performed with a custom-built epifluorescence microscope with pulsed laser and microwave interrogation, shown in Fig. 1b (see Supplementary Note 2). Using ~140 mW of 520 nm laser light over a 25-µm-diameter spot fluorescence from ≳10 million NV centers adjacent to ~1 pl of analyte was detected. The analyte volume reported here is the physical volume the solution occupies within the laser spot, which is given by the product of the grating height (~3 μm), grating duty cycle (~0.5), and laser spot area π × (25 μm/2)^2^, or 0.7 pl. A static magnetic field *B*_0_ = 20–50 mT was applied along one of the four NV axes with a permanent magnet, and the aligned NV sub-ensemble was optically interrogated using resonant microwaves (10–15 MHz Rabi frequency) delivered by a copper loop fabricated on a cover slip.

To assess the anticipated improvement of the nanograting chips, the nanograting sidewalls and the flat surfaces of unstructured diamond chips were doped at similar conditions to have the same nitrogen density (Fig. 2d, *inset*). Figure 2d, e compares the NV transverse spin coherence time *T*_2_ (measured with the XY8-22 pulse sequence^31^) and fluorescence intensity for flat and nanograting chips implanted at different energies. While *T*_2_ for flat and nanograting chips is approximately the same (and is similar to *T*_2_ for normal-incidence high-dose implantation^13^), the fluorescence intensity is 20–50 times brighter for nanograting chips. We attribute this to the 2/tan 4° = 28 times higher effective dose captured by the nanograting chip and improved collection efficiency from nanostructures^32^. These results highlight the advantage of nanogratings in NMR sensitivity, since fluorescence intensity is proportional to *ηN*_NV_, whereas ${\rho _{{\rm{min}}}} \propto 1{\rm{/}}\sqrt {\eta {N_{_{{\rm{NV}}}}}}$. The nanostructuring process does not negatively affect other parameters in Eq. (1). The fluorescence contrast of Rabi oscillations was 2.7 ± 0.8% for all studied diamond chips independent of nanofabrication or doping parameters.

### NV NMR spectroscopy

We used a correlation spectroscopy pulse sequence for NV NMR detection of external solutions^33^, as described in Fig. 3a. This sequence correlates the nuclear magnetic fields at different points in time, encoding this information in the NV spin state and corresponding fluorescence intensity. Specifically, we used two XY8-N pulse trains, both tuned to the target nuclear Larmor frequency and separated by a variable delay $\tilde \tau$. As $\tilde \tau$ is swept, the relative NV fluorescence intensity, Δ*F*/*F*, oscillates at the nuclear Larmor frequency, analogous to a nuclear free induction decay (FID). The Fourier transform of this FID-like signal reveals the NMR spectrum, from which we extract the spin density.

Figure 3b, c shows the time-domain and frequency-domain NMR signals for ^19^F nuclei in Fomblin^®^ oil (6600 Da) using the 20 keV nanograting chip. The sensor response was converted to absolute units of nT^2^ using analytical expressions (see Supplementary Note 1) that were validated using calibrated magnetic fields from a test coil. To confirm that the signals arise from the target nuclei, we repeated these measurements at different magnetic fields and with ^1^H-rich analytes (glycerol and immersion oil). The resulting NMR peaks were always at the expected Larmor frequency of each species (Fig. 3d), and NMR peaks were either absent (for ^19^F) or greatly diminished (for ^1^H) when the analyte was removed^13, 14^.

**Fig. 4:**
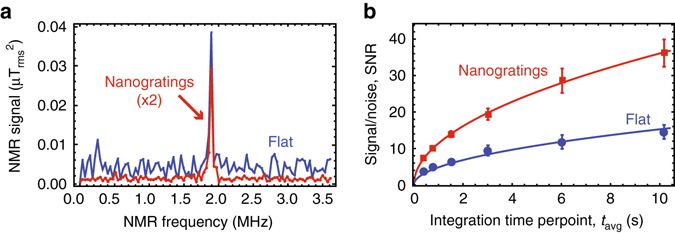
NV NMR sensitivity characterization. **a** ^19^F NMR signal from Fomblin^®^ oil at *B*_0_ = 47.1 mT for flat and nanograting sensors implanted at 20 keV using XY8-13 correlation sequence. **b** NMR signal-to-noise ratio as a function of averaging time. Fits to the function SNR = $\alpha \sqrt {{t_{{\rm{avg}}}}}$ give good agreement for both sensors (*solid lines*). The coefficient *α* was 2.4 times larger for the nanograting chip. *Error bars* represent standard error of the mean

To characterize the spin-concentration sensitivity, we continuously acquired NMR spectra of ^19^F nuclei in Fomblin^®^ oil and compared SNR for both flat and nanograting sensors implanted at 20 keV. Figure 4a shows characteristic NMR spectra for each. The nanograting-sensor noise, defined as the standard deviation of points adjacent to the NMR peak, is a factor of 6 smaller than the flat sensor noise. This is primarily due to the ~20× larger fluorescence intensity (Fig. 2e) exhibited by the nanograting sensor, which leads to smaller relative photon shot noise. However, the nanograting sensor signal strength, defined as the ^19^F NMR peak amplitude in nT^2^, is ~2.5× smaller. This was unexpected; the signal amplitude should only depend on the NV depth, which should be the same for both sensors under identical implantation conditions (Fig. 2d, *inset*). A likely cause for this discrepancy is that the tops of the nanogratings were inadvertently implanted because of degradation of the etch mask (Fig. 2a). Ions bombarding the nanograting tops are nearly normally incident to the surface, resulting in NV centers that are too deep to sense external nuclei, reducing the overall NMR signal contrast. If 50% of the NV centers were formed from deep implantation into the nanograting tops, we would expect a twofold reduction in signal. Other contributions may be from deep implantation into the flat bottom of the nanogratings or imperfect wetting.

Regardless, the nanograting sensors had better overall SNR. To acquire each spectrum, $\tilde \tau$ was swept and the signal was averaged for a variable time *t*_avg_. Figure 4b plots the SNR as a function of *t*_avg_, revealing a 2.4× SNR improvement with the nanogratings. Fomblin^®^ has 40 ± 2 × 10^24 19^F spins per liter, and we detect it with SNR = 11.4 ± 0.2 at *t*_avg_ = 1 s. We thus determine a minimum detectable concentration *ρ*_min_ = 11 ± 1 × 10^24^ spins per liter. Throughout we use *t*_avg_ as the effective integration time, since the number of points used to generate the spectrum may be reduced using optimized sampling strategies^34^.

Next, we performed NMR spectroscopy on more dilute solutions to demonstrate our capability. We selected CsF dissolved in glycerol as our test analyte. Glycerol was chosen as the solvent because of its high viscosity, which limits molecular diffusion, while CsF was selected as the target molecule because of its high solubility in glycerol and because ^19^F has the second largest magnetic moment among common nuclear isotopes. Protons have a larger magnetic moment but were not suitable because a background proton signal was often present^13, 14^. Using a 20% CsF/glycerol solution by weight, the ^19^F concentration is 1.0 × 10^24^ spins per liter, ~40 times lower than Fomblin^®^. To successfully obtain a spectrum with the same SNR requires ~1600 times more signal averaging, a task previously not practical in NV NMR.

**Fig. 5:**
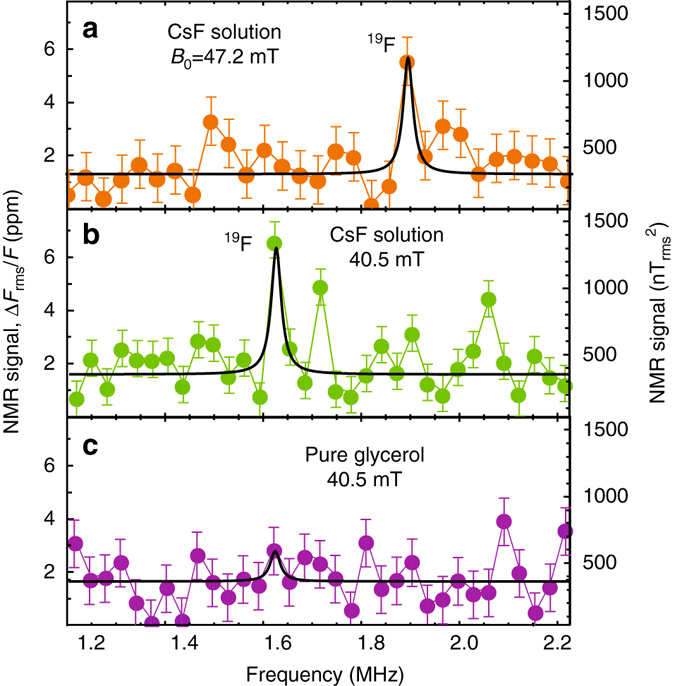
Solution NMR. **a**, **b** NMR spectra of 20% by weight CsF solution in glycerol at 47.2 and 40.5 mT. **c** NMR spectrum of pure glycerol. All spectra were measured with an XY8-10 correlation spectroscopy pulse sequence tuned to the ^19^F precession frequency. Each spectrum was fit to a single Lorentzian function with central frequency set to the anticipated ^19^F Larmor frequency. The *linewidth* was fixed to that of the ^1^H linewidth in glycerol obtained under similar conditions. The only free fit parameters were peak amplitude and offset. We assume that each spectrum contains at most one NMR line (from ^19^F) and other fluctuations are due to noise. *Error bars* represent standard deviation of off-resonant points

Figure 5 shows NMR spectra for this solution. At *B*_0_ = 47.2 mT, we observe a peak at the ^19^F Larmor frequency. When changing the magnetic field to *B*_0_ = 40.5 mT, the peak moves according to the ^19^F gyromagnetic ratio and maintains a comparable amplitude. Finally, when the analyte is replaced with pure glycerol the peak disappears, as expected. For the ^19^F peak, the SNR is 4 ± 1 at *t*_avg_ = 77 s, which corresponds to a minimum detectable concentration *ρ*_min_ = 6 ± 2 × 10^24^ spins per liter. This is roughly a factor of 2 better than for Fomblin^®^ measurements due to more optimal readout timing.

## Discussion

**Fig. 6:**
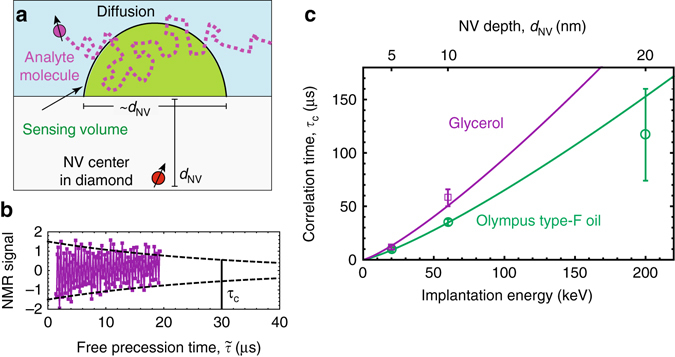
Diffusion-limited NMR. **a** Diffusion of analyte molecules through the sensing volume. **b** Example of temporal decay of the correlation signal for immersion oil. The decay is exponential with a characteristic correlation time *τ*_C_. *Dashed line* represents the decay envelope. **c** Correlation time *τ*_C_ as a function of nitrogen implantation energy for immersion oil and glycerol. *Error bars* represent fit uncertainty. *Solid lines* are fits to the one-sided diffusion model discussed in the text

The present platform still has significant room for improvement in sensitivity and spectral resolution. The observed sensitivity is about two orders of magnitude worse than the theoretical sensitivity calculated with Eq. (1) and plotted in Fig. 1a. The largest contributions to this discrepancy are (see Supplementary Note 1): NV control pulse errors^35^, which reduce the contrast by ~5× from the ideal case; imperfect doping parameters, which reduce *N*_NV_ by up to 5× and increase *d*_NV_ by up to 2× from the ideal case; and the previously discussed ~2.5× lower signal strength for nanograting samples. The spectral resolution, which is inversely proportional to the analyte correlation time *τ*_C_, is currently limited to a few kHz. For nanoscale sensing volumes, analyte molecules rapidly diffuse across the sensing region (Fig. 6a), limiting *τ*_C_. Figure 6b plots a typical correlation signal that decays exponentially with time constant, *τ*_C_, in the microsecond range. Figure 6c plots *τ*_C_ of protons in immersion oil and glycerol as a function of NV layer depth *d*_NV_. The data are fit with a one-sided diffusion model^36^, ${\tau _{\rm{C}}} = 2d_{_{{\rm{NV}}}}^2{\rm{/}}D$, where *D* is the molecular diffusion coefficient, revealing *D*_glyc_ = 4.4 ± 0.6 × 10^−12^ m^2^/s and *D*_oil_ = 6.4 ± 0.3 × 10^−12^ m^2^/s. These values are slightly higher than room-temperature literature values (*D*_glyc_ = 2.5 × 10^−12^ m^2^/s and *D*_oil_ = 0.5–2.5 × 10^−12^ m^2^/s)^36, 37, 38^, which may be attributed to elevated analyte temperature or hygroscopic effects. The qualitative agreement supports the hypothesis that diffusion is responsible for the short correlation times and improves confidence in the NV depths reported by simulations. Lowering the temperature or using microporous media^39^ would restrict translational diffusion, improving the resolution and *ρ*_min_, although dipolar broadening may be a limitation. Alternatively, the use of dynamic nuclear polarization may enable NV NMR with nuclear-*T*_1_-limited resolution and improved sensitivity due to coherent nuclear precession^40, 41^.

Nevertheless, the current platform’s record sensitivity makes it promising for solid-state NMR and nuclear quadrupole resonance spectroscopy of trace powders and thin films^42, 43^, since these applications require only coarse frequency resolution and diffusion is restricted. It may also find application in NMR relaxometry^44^ and solution NMR for impurity profiling and quality control of pharmaceuticals^45^. These applications could benefit from the ease of microfluidic integration, which would permit parallel measurements. Future implementations may also harness the optical waveguiding properties of the nanogratings to reduce excitation intensity and increase fluorescence collection by exciting and collecting light through the sides of the chip.

In summary, we performed NMR spectroscopy of picoliter-volume solutions using a nanostructured diamond-chip platform. The sensor uses non-inductive detection of statistical polarization, avoiding the need for large magnetic fields or hyperpolarization. Etching dense, high-aspect-ratio nanogratings into the diamond surface resulted in a 15-fold improvement in surface area and more than 20-fold increase in fluorescence intensity without sacrificing the NV spin properties. Using a solution of CsF in glycerol, we determined 4 ± 2 × 10^12^ spins in an ~1 pL volume can be detected with a SNR of 3 in 1 s integration. This concentration sensitivity was independently confirmed using Fomblin oil and found to agree to within a factor of 2. This represents nearly two orders of magnitude improvement over previous picoliter NMR demonstrations.

## Methods

### Fabrication details

Nanogratings were fabricated on the surfaces of electronic-grade diamond chips purchased from Element 6 (initial dimensions 2 × 2 × 0.5 mm^3^, 1.1% ^13^C abundance, [100]-polished faces, [110] sides). After cleaning the chips for 7 h in a 1:1:1.3 mixture of nitric:perchloric:sulfuric acids at 200 °C (from now on referred to as triacid cleaning), each diamond chip was mounted flat on a silicon substrate. The chip was surrounded by four 0.5-mm-thick stainless-steel sheets flush with the diamond surface to avoid edge beading during spin coating. A thin film of i-CON 16 was used as an adhesive to stick the diamond and the steel sheets to the silicon substrate because it does not outgas or reflow during baking. The next step was to spin-coat i-CON 16 as an anti-reflection coating (ARC) on top of the diamond (4000 rpm, 150 °C oven bake for 5 min), followed by spin-coating with a ultraviolet (UV) negative photoresist (NR-7 500p, 4000 rpm, 150 °C hot plate bake for 1 min).

Interferometric lithography^26, 27^ was used to produce optical standing waves with a period of ~400 nm. With interferometric lithography, the entire diamond surface was covered with periodic nanostructures in a few seconds of exposure time. By varying the resist, postbake, and development conditions, the grating duty cycle can span at least 20–80%. Most of the samples used in this work had ~50% duty cycle. After developing for 1 min in RD6 developer, the un-masked regions of ARC were removed using a reactive ion etch (10 sccm O_2_ flow, 10 W radio frequency power) for 1 min. For the metal mask, 3–5 nm of Cr (used for adhesion) followed by 70 nm of gold were deposited on the masked diamond chips. Liftoff was performed by immersing the chips in a sonicating acetone bath for 10 min to leave only Cr/Au grating structures on the diamond surface. Next, a highly anisotropic oxygen/argon etch using inductively coupled plasma (ICP; 8 sccm Ar_2_ flow, 16 sccm O_2_ flow, 30 W forward radio frequency power, 450 W ICP radio frequency power) was applied for 40 min to form nanogratings up to 3 μm deep.

In the final etch step, there is a tradeoff between etching deeper and losing the mask. Ideally, the mask would remain thick enough (≳10 nm) to block ions from penetrating the tops of the nanogratings during implantation. This is because ions incident on the nanograting tops would travel on average approximately six times deeper than those incident to the sidewalls due to the large difference in angles of incidence. At such a large depth these NV centers will hardly register any NMR signal, reducing the overall NMR signal contrast. We found that the 40 min of etching used in this work was longer than optimal, and the mask had eroded over large regions of the chips prior to implantation. We have since optimized the process to use a slightly thicker metal mask such that the mask remains largely intact after 35 min of etching.

After fabrication, the nanograting sidewalls were implanted with nitrogen. When possible, we left the remaining metal mask used for etching to block ions from penetrating deep into the tops of the gratings. Implantation was performed by Materials Diagnostics (Albany, NY) using a ^15^N^+^ ion beam with 20–200 keV implantation energy (corresponding to ~5–20 nm typical NV depth), delivering a dose of 2 × 10^13^ to 8 × 10^13 15^N^+^ ions/cm^2^ at 4 degree implantation angles (Supplementary Note 2). For comparison, flat chips were mounted vertically (to implant at the same angle; see Fig. 2d, *inset*). Following implantation, the chips were cleaned in triacid overnight, then annealed in vacuum in a multistep annealing process (800 °C for 4 h followed by 1100 °C for 2 h) to form an NV layer at the nanograting sidewall surfaces. This annealing procedure was selected to give a high NV^−^ yield while minimizing the abundance of other paramagnetic impurities^46^. After annealing, the diamond chips were again cleaned in triacid for 10 h to remove surface residues and mounted in the microscope set-up. Triacid treatment is expected to yield a hydrophilic, oxygen-terminated surface. Although the triacid treatment proved sufficient for wetting and NV properties, other treatments may provide additional advantages^47–49^. Each time we removed a diamond chip from the set-up, we used the same acid-washing procedure to remove immersion oil, Fomblin oil, CsF/glycerol solutions, or other contaminations.

### Experimental procedure

After mounting a diamond chip in the microscope, we align the *B*_0_ field from a permanent magnet by measuring the optically detected magnetic resonance (ODMR) frequencies of the four NV orientations. Good field alignment is important because *T*_2_ is maximized and the NV contrast is maximized when *B*_0_ is aligned along the N–V axis^50^, resulting in better sensitivity. We use the NV sub-ensemble aligned with *B*_0_ for correlation spectroscopy, while the other three NV sub-ensembles do not participate in the measurement and contribute background fluorescence. After aligning, we calculate the *B*_0_ magnitude from the ODMR spectrum. We position the microwave wire to achieve a reasonably fast Rabi frequency (10–15 MHz) and test the correlation spectroscopy experiment with the AC magnetic field from a calibrated test coil (driven by a sine wave from a function generator).

When performing NMR spectroscopy, we select parameters for the correlation pulse sequence based on the following principles. Laser light pulses have 5 μs duration. The first ~1 μs is used for readout, while the remainder is used to efficiently repolarize the NV centers. The separation between *π*-pulses, 2*τ*, is chosen to match half the nuclear Larmor period of the target spin, 4*τ* = *τ*_L_. We select the number of repetitions (the “N” in XY8-N) which produces the highest signal-to-noise-ratio (SNR), at constant measurement time, using protons in pure glycerol as a convenient test sample. The step size between $\tilde \tau$ values is chosen to be ~*τ*_L_/4, and the longest value is chosen to match the approximate duration of the XY8-N sequences. This represents a compromise between obtaining high SNR (which would seek to minimize the overall measurement time) and obtaining high spectral resolution (which would prefer to use as long a $\tilde \tau$ as possible). In every experiment we alternate the phase of the final *π*/2-pulse and subtract the fluorescence signals, resulting in fast common-mode rejection of fluorescence intensity drifts.

We have noticed that the fluorescence intensity depends on the time between laser pulses in a non-trivial manner, presumably due to complex NV^0^/NV^−^ dynamics. To circumvent related systematic effects, we add buffer time between the last microwave *π*/2-pulse and the laser readout pulse to ensure that the time between laser pulses remains constant while we sweep $\tilde \tau$. However, this means most experiments take ~1.5× longer than they should because of the buffer time. We average fluorescence readout time traces on an oscilloscope, which introduces additional dead time. The oscilloscope misses triggers while averaging and data processing, and it also spends time transferring averaged time traces to the acquisition computer. These dead times collectively make the actual experiments roughly ~2.5× slower than necessary, which we will improve in future setups. When determining *t*_avg_ in the main text, we neglect this dead time.

### Data availability

The data used to generate the figures in this work are available from the corresponding author upon request.

## Electronic supplementary material

Supplementary Information

## Electronic supplementary material

**Supplementary Information** accompanies this paper at Doi: 10.1038/s41467-017-00266-4.
