# Supplementary material for: Solution nuclear magnetic resonance spectroscopy on a nanostructured diamond chip
Source: Nat Commun. 2017 Aug 4;8:188. doi: 10.1038/s41467-017-00266-4 (PMC5543112; doi:10.1038/s41467-017-00266-4)
Supplement: Supplementary file 1 — Supplementary Information [file 41467_2017_266_MOESM1_ESM.pdf]

File name: Supplementary Information
Description: Supplementary Figures, Supplementary Notes, Supplementary Tables and Supplementary References.

SUPPLEMENTARY NOTE 1
SENSITIVITY ASSESSMENT

Thermal and statistical polarization comparison

In the case where $h\gamma_{\mathrm{nucl}}B_0 \ll k_{\mathrm{B}}T$ (the Curie's law high-temperature limit), where B_0 is the applied field, $k_{\mathrm{B}} = 1.381 \times 10^{-23}$ J/K is the Boltzmann constant, and T is the temperature, the field from nuclear thermal polarization is (ignoring numerical factors of order 1 from the discrete nuclear spatial distribution):

$$B_{\mathrm{therm}} \sim \frac{\mu_0}{4\pi}\frac{h^2\gamma_{\mathrm{nucl}}^2 B_0\rho}{4k_{\mathrm{B}}T}, \tag{1}$$

where $\mu_0 = 4\pi \times 10^{-7}$ m·T/A is the vacuum permeability, $h = 6.626 \times 10^{-34}$ J·s is the Planck constant, γ_{nucl} is the nuclear gyromagnetic ratio in MHz/T (42.58 MHz/T for ^{1}H), and ρ is the nuclear spin number density. This field is roughly constant for a sample-sensor distance of order the thickness of the analyte after which it falls off as the distance cubed.

In contrast, NV centers near a flat diamond surface experience a magnetic field from the statistical polarization of the nuclei. Averaged over time (and over the NV ensemble), the field magnitude approaches the standard deviation of the normally-distributed random magnetization from configurations of spins within each NV center's detection volume. The field component along the N-V axis from the nuclear statistical polarization, B_{RMS}, is given by [1]:

$$B_{\mathrm{RMS}}^2 = \frac{\pi(8 - 3\sin(\alpha)^4)}{128}\left(\frac{\mu_0 h\gamma_{\mathrm{nucl}}}{4\pi}\right)^2\frac{\rho}{d_{\mathrm{NV}}^3} = P(\alpha)\left(\frac{\mu_0 h\gamma_{\mathrm{nucl}}}{4\pi}\right)^2\frac{\rho}{d_{\mathrm{NV}}^3}, \tag{2}$$

where d_{NV} is the NV depth below the diamond surface. The function $P(\alpha) = \frac{\pi(8-3\sin(\alpha)^4)}{128}$ is a geometric factor that comes from the angle α the N-V axis makes with the normal to the substrate surface (see Fig. 6 in Ref. [1]). Some previous experiments used flat diamond chips polished along the [100] crystallographic plane. The flat samples in the present manuscript also use this geometry. In this case, all four NV orientations have the same $\alpha = \cos^{-1}(\sqrt{1/3}) = 54.7°$ angle to the surface. The grating sidewalls in our nanograting chips are approximately along the [110] crystallographic direction. Two of the NV orientations lie in-plane (with slightly worse sensitivity than with [100]) and the other two orientations lie out-of-plane (with slightly better sensitivity). Supplementary Table 1 details $P(\alpha)$ for these geometries.

Diamond geometry	Surface	$\sin(\alpha)$	$P(\alpha)$
Flat diamond	[100]	$\sqrt{\frac{2}{3}}$	$\frac{5\pi}{96} \approx 0.052\pi$
Grating (out-of-plane)	[110]	$\sqrt{\frac{1}{3}}$	$\frac{23\pi}{384} \approx 0.060\pi$
Grating (in-plane)	[110]	1	$\frac{5\pi}{128} \approx 0.039\pi$

Supplementary Table 1. **Example $P(\alpha)$ values.** A table listing $P(\alpha)$ for relevant NV pointing directions, which give rise to different B_{RMS}^2. The NVs in our nanograting sidewall surfaces have two angles with respect to the sidewall normal direction, one of which is ∼50% more sensitive to the nuclear statistical polarization than the other.

Supplementary Fig. 1(a) compares the ^{1}H statistical and thermal fields as a function of water volume, V. For thermal polarization, the field is independent of sample volume provided that the NV centers are located sufficiently close ($\lesssim V^{1/3}$). For statistical polarization, the field is a function of the NV depth, d_{NV}. Intuitively this is because the technique relies on the random net difference

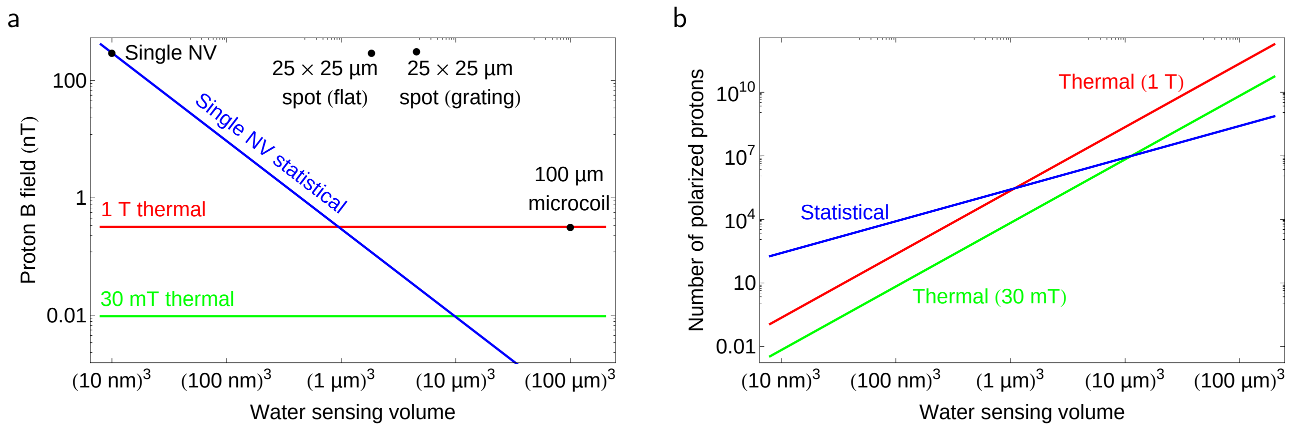

Supplementary Figure 1. **Volume dependence of thermal and statistical polarization.** Comparison between thermal and statistical nuclear polarization as a function of detection volume for ^{1}H nuclei in water. (a) The typical magnetic field from thermally-polarized nuclei (red and green lines) depends linearly on B_0 but is independent of sample volume. The statistical magnetic field is independent of B_0, but for a single NV (blue line) it falls off rapidly with detection volume since that requires a greater NV depth. Using an NV ensemble with a constant depth increases the sensing volume without sacrificing the field strength. Here we plot the expected B_{RMS} for 10 nm deep NV ensembles in a (25 µm)^2 spot for flat and grating chips. The volume for a flat chip is not practical for fluids, as it is only 10 nm thick. However the volume for the nanogratings case is realistic; it only assumes the nanograting grooves are filled to the top as in the present experiments. (b) The number of thermally-polarized nuclei increases linearly with V while the number of statistically-polarized nuclei increases like $\sqrt{V}$.

in magnetization expressed as a fraction. For small ensembles of nuclei this fraction is much larger, producing a correspondingly larger magnetic field. Assuming the NV statistical sensing volume is $V \approx d_{\text{NV}}^3$, the field scales as $V^{-1/2}$.

Supplementary Fig. 1(b) compares the number of polarized spins which contribute to an NMR signal for thermal and statistical polarization detection. For spin-1/2 nuclei in a detection volume V, there are $N_{\text{therm}} = \frac{h\gamma_{\text{nucl}} B_0}{2k_{\text{B}}T}\rho V$ thermally-polarized nuclei and $N_{\text{stat}} = \sqrt{\rho V}$ statistically-polarized nuclei [2]. For sufficiently small B_0 and V, the statistical polarization dominates over thermal. An additional advantage is the correlation spectroscopy technique used here does not require radio frequency (RF) pulses to control the nuclei, avoiding the need for high RF power to manipulate nuclear spins and allowing for a broader NMR spectral bandwidth. Moreover, since the nuclei are never manipulated (and are therefore always in equilibrium), there is never a need to wait several nuclear longitudinal spin relaxation times (T_1) before starting a new experiment. This is particularly advantageous if the nuclear T_1 is much longer than the inhomogeneously-broadened nuclear transverse spin relaxation time T_2^*, where a duty cycle of order T_2^*/T_1 would be required in conventional NMR.

Theoretical nuclear concentration sensitivity

In correlation spectroscopy, the nuclear Larmor precession is encoded as oscillations in the NV spin populations and resulting fluorescence intensity. Following the analysis in Ref. [3] for correlation spectroscopy using Hahn echo, we treat the nuclear statistical magnetization as an AC magnetic field (Larmor frequency f_{nucl}) with a random amplitude that is normally distributed with a variance

$B^2_{\rm RMS}$. The correlation time of the nuclear spins is presumed to be sufficiently long that the nuclear field is constant throughout a single pulse sequence but short enough that all possible amplitudes are averaged over many experiments. Assuming the NV centers experience a small statistical nuclear field, $B_{\rm RMS} \ll (4\gamma_{\rm NV}\tau_{\rm L})^{-1}$, the NV fluorescence intensity after a correlation spectroscopy pulse sequence is:

$$F = N_{\rm phot}\left(1-\frac{C}{2}\right) + \frac{N_{\rm phot}C}{2} \times \frac{1}{2}\left[\frac{4\gamma_{\rm NV}B_{\rm RMS}}{f_{\rm nucl}}\sin^2\left(\frac{2\pi f_{\rm nucl}\tau}{2}\right)\right]^2 \cos\left(2\pi f_{\rm nucl}(2\tau+\tilde{\tau})\right). \quad (3)$$

Here $N_{\rm phot}$ is the number of photons collected in one readout, C is the correlation spectroscopy fluorescence contrast (see Supplementary Fig. S1 in Ref. [3]), $\gamma_{\rm NV} = 28.03$ GHz/T is the NV gyromagnetic ratio, $f_{\rm nucl} = \gamma_{\rm nucl}B_0$ is the nuclear Larmor frequency, τ is the time duration between the NV $\pi/2$-pulse and π-pulse, and $\tilde{\tau}$ is the free precession time between the two Hahn echo sequences. In a Hahn-echo correlation sequence, we tune the pulse spacing to match the nuclear Larmor frequency such that $2\tau = \tau_{\rm L}$ and replace $f_{\rm nucl}$ with $1/T_{\rm tot}$, where $T_{\rm tot}$ is the total phase accumulation time. This simplifies the expression for fluorescence intensity to

$$F = N_{\rm phot}\left(1-\frac{C}{2}\right) + 4N_{\rm phot}C\left(\gamma_{\rm NV}B_{\rm RMS}T_{\rm tot}\right)^2\cos\left(2\pi f_{\rm nucl}\tilde{\tau}\right). \quad (4)$$

This expression also holds for the more complicated XY8-N sequence. The primary difference is the phase accumulation time, $T_{\rm tot}$, is a factor 4N longer in the XY8-N sequence as compared to the Hahn echo version.

If the measurement is photon shot-noise-limited, then $\Delta F = \sqrt{N_{\rm phot}}(1-C/2) \approx \sqrt{N_{\rm phot}}$, and the minimum detectable field strength (SNR=1) is given by:

$$\Delta B^2_{\rm RMS,min} = \frac{\Delta F}{|\partial F/\partial B^2_{\rm RMS}|} = \frac{1}{4\sqrt{N_{\rm phot}}C(\gamma_{\rm NV}T_{\rm tot})^2}. \quad (5)$$

Combining Supplementary Eq. 2 and Supplementary Eq. 5 yields $\rho_{\rm min}$, the minimum-detectable magnetic spin concentration, after one pulse sequence (substituting $h = 2\pi\hbar$):

$$\rho_{\rm min}({\rm SNR}=1) = \frac{1}{P(\alpha)(\mu_0\hbar\gamma_{\rm NV}\gamma_{\rm nucl})^2} \times \frac{d^3_{\rm NV}}{T^2_{\rm tot}C\sqrt{N_{\rm phot}}}. \quad (6)$$

To evaluate the minimum-detectable concentration after one second of averaging, we divide by $\sqrt{N_{\rm r}}$, where $N_{\rm r}$ is the number of readouts per second. We also replace $N_{\rm phot}$ with the product of the number of NVs ($N_{\rm NV}$) and the number of photons collected per NV per readout (η). Imposing the NMR standard requirement of SNR = 3 in 1 s, we obtain:

$$\rho_{\rm min}({\rm SNR}=3 \text{ in } 1\text{ s}) = \frac{3}{P(\alpha)(\mu_0\hbar\gamma_{\rm NV}\gamma_{\rm nucl})^2} \times \frac{d^3_{\rm NV}}{T^2_{\rm tot}C\sqrt{\eta N_{\rm NV}N_{\rm r}}}. \quad (7)$$

In Fig. 1(a) of the main text we plot $\rho_{\rm min}({\rm SNR}=3 \text{ in } 1\text{ s})$ as a function of analyte volume using the following parameters: $\gamma_{\rm nucl} = 40.08$ MHz/T, $P(\alpha) = 23\pi/384$ (Supplementary Table 1), $d_{\rm NV} = 5$ nm, $T_{\rm tot} = 25$ µs, $C = 0.02$, $N_{\rm r} = 2\times10^4$, and $\eta = 0.03$. To determine how $N_{\rm NV}$ scales with volume, we assume that a dose of 5×10^{13} $^{15}{\rm N}^+/{\rm cm}^2$ is delivered to the chip, and, after annealing, the nitrogen-to-NV conversion efficiency is 10% [4], resulting in 5×10^4 ${\rm NV}^-/\mu{\rm m}^2$. Thus $N_{\rm NV}$ scales linearly with sensor area. We then assume the gratings are 2 µm tall with 50% duty cycle and that analyte fills the gratings flush to the top (i.e. there is no gap between coverslip and nanogratings). In this case, the analyte volume also scales linearly with sensor area. Combining

with Supplementary Eq. (7), we find that the minimum detectable concentration $\rho_{\text{min}} \propto \text{volume}^{-1/2}$ as seen in Fig. 1(a) of the main text. For 1 pL of analyte, $N_{\text{NV}} \approx 5 \times 10^7$; for 1 nL of analyte, $N_{\text{NV}} = 5 \times 10^{10}$, etc.

Using these theoretical values, we determine $\rho_{\text{min,theory}} \approx 4 \times 10^{22}$ spins/L for the ∼1 pL analyte volume used in this work. This concentration sensitivity represents a giant improvement over previous small-volume NMR works; it is comparable to microslot NMR at high field, but with a 4-orders-of-magnitude smaller volume. While our experimental values for ρ_{min} (6×10^{24} spins/L with Fomblin®) still represent a large improvement over previous work, they are about 150 times larger than this theoretical estimate. The experimental parameters are consistent with $T_{\text{tot}} \approx 25$ µs and $N_{\text{r}} \approx 2 \times 10^4$ (when dead time is excluded), but the experimental fluorescence levels ($\propto \eta N_{\text{NV}}$) are about 5× lower than expected from the parameters in the calculation, likely due to suboptimal N-to-NV conversion efficiency. An even larger deviation between experiment and theory is the experimental contrast, $C \approx 0.004$, which is approximately 5 times lower than ideal. We attribute this to pulse errors, as C decreases when decreasing the Rabi frequency or increasing the number of pulses used in the sequence [5]. Another deviation from theory is the ∼2.5× reduction in signal between flat and nanostructured samples discussed in the text (presumably due to deep implantation of nanograting tops/bottoms and imperfect wetting).

The above three factors account for ∼28× difference in ρ_{min} between theory and experiment. The remaining factor of ∼5 appears to be due to an over-estimation of the expected field strength from the analyte. From Supplementary Eq. (2), the field strength for Fomblin is given by $B^2_{\text{RMS,theory}} = 0.37\ \mu\text{T}^2$ at an NV depth $d_{\text{NV}} = 5$ nm. This is ∼9 times larger than the observed value in Fig. 4(a) in the main text, and largely accounts for the remaining observed discrepancy in ρ_{min}. This over-estimation suggests the chips have a deeper characteristic NV depth than expected. Possible reasons include a deviation in NV depth profile from SRIM, the presence of a few-nm surface layer of water or hydrocarbons, and/or the presence of debris or other interface issues that reduce the overall sensor-sample contact. If instead we use $d_{\text{NV}} = 10$ nm, we find $B^2_{\text{RMS,theory}} = 0.04\ \mu\text{T}^2$, identical to the observed value in Fig. 4(a). Note that if the characteristic NV depth is ∼2× deeper than expected, it would reduce the fitted diffusion constants in Fig. 6 of the main text by a factor of ∼4. In future work we will explore lower-energy implantation to reduce d_{NV}. Finally, we note that there is uncertainty in our estimation of correlation spectroscopy contrast C. We can not rule out that some of the remaining factor of ∼5 discrepancy is partly due to imprecision in this estimate.

Concentration sensitivity assessment

Before settling on CsF in glycerol, we considered several analytes and target spin species to characterize our spin concentration sensitivity ρ_{min}. A major requirement was that the analyte have a sparser concentration than those used in previous NV and picoliter NMR work. For example, it should be smaller than the proton density in water, which is 6.7×10^{25} spins/L = 110 M). Supplementary Table **2** lists example nuclear densities (top half) and some candidate solutions (bottom half). Ideally we want a viscous solvent with slow molecular diffusion D to maximize the nuclear correlation time τ_{C}. Glycerol ($C_3H_8O_3$) is a promising solvent choice because it is viscous and is a common solvent, dissolving many different molecules. Although ^{1}H is a desirable nucleus to detect (γ_{nucl} is large and many chemicals contain hydrogen), we avoided using it because diamond surfaces can have a ∼1 nm adsorbed water or hydrocarbon layer, which produces a large background proton signal even in the absence of analyte [6]. Furthermore, the baseline ^{1}H concentration in the commonly-available deuterated glycerol (glycerol-d_8, 98% deuterated) is comparable to or larger than the saturation concentration of common analytes like sucrose ($C_{12}H_{22}O_{11}$). We therefore restricted our search to analytes featuring NMR-active nuclei other than protons.

The bottom half of Supplementary Table 2 considers alternative solutions and target nuclei. The figure of merit is $B^2_{\rm RMS} \propto \rho\gamma^2_{\rm nucl}$ from Supplementary Eq. 2, so we considered nuclei with large $\gamma_{\rm nucl}$. Although $B^2_{\rm RMS}$ for saturated solutions of Na_2CO_3 is slightly larger than for CsF, ^{23}Na is spectrally difficult to distinguish from ^{13}C internal to the naturally-abundant diamond chips. For NMR peaks to be distinguishable their linewidth must be smaller than $1/\tau_C \approx 100$ kHz. For peaks due to ^{23}Na and ^{13}C, this becomes feasible only at $B_0 \gtrsim 180$ mT, which was out of the range studied here. Thus, we chose CsF in glycerol. The CsF saturation concentration was unavailable in the literature, but we determined it to be between 20-40% by weight by varying the CsF concentration and monitoring precipitate levels.

Spin/analyte	$\gamma_{\rm nucl}$ (MHz/T)	Abundance	ρ (spins/L)	Comments
^{1}H in H_2O	42.58	100%	6.7×10^{25}	
^{1}H in IMMOIL-F30CC oil	42.58	100%	6×10^{25}	[7]
^{1}H in glycerol	42.58	100%	6.6×10^{25}	
^{13}C in glycerol	10.71	1.1%	2.7×10^{23}	
^{1}H in 98% glycerol-d$_8$	42.58	2%	1.4×10^{24}	Sigma-Aldrich 447498
^{1}H; saturated sucrose in 98% glycerol-d$_8$	42.58	100%	2.6×10^{24}	Ignores ^{1}H from above line [8]
^{19}F in Fomblin®	40.08	100%	4.0×10^{25}	Sigma-Aldrich 317993
^{13}C in diamond crystal	10.71	1.1%	1.9×10^{24}	Spins fixed in crystal lattice
^{19}F; 0.2 g CsF in 1 g glycerol	40.08	100%	1.0×10^{24}	What we used ($\star$)
^{23}Na; saturated NaCl in glycerol	11.27	100%	1.1×10^{24}	12× weaker $B^2_{\rm RMS}$ than $\star$ [9]
^{23}Na; saturated Na_2CO_3 in glycerol	11.27	100%	1.4×10^{25}	1.1× stronger $B^2_{\rm RMS}$ than $\star$ [10]
^{11}B; saturated $B(OH)_3$ in glycerol	13.66	80.1%	3.0×10^{24}	3.5× weaker $B^2_{\rm RMS}$ than $\star$ [10]

Supplementary Table 2. **Target analyte $B^2_{\rm RMS}$ evaluation.** Comparison of analytes with different nuclear spin densities. The top half includes relevant reference nuclear densities, and the bottom half lists solutions we considered for testing. Note that the solution volume is slightly larger than the original solvent volume after adding a solute, but for simplicity we neglect this typically small effect. We also assume that the solute molecular diffusion constant D is the same as the self-diffusion of glycerol, and that the diffusion is unaffected by dissolving solutes.

Optimal NV layer depth

Supplementary Eq. (7) provides insight on how to optimize the NV layer depth, $d_{\rm NV}$, to achieve the smallest detectable $\rho_{\rm min}$ in the limit of fast molecular diffusion and shallow NVs. According to Supplementary Eq. (7), in a correlation spectroscopy measurement, $\rho_{\rm min} \propto d^3_{\rm NV}/(T^2_{\rm tot}\sqrt{N_{\rm r}})$. If the XY8-N sequences dominate the correlation experiment duration, then $N_{\rm r} \propto 1/T_{\rm tot}$ since fewer experiments can be done in 1 second if the duration is longer. Thus, $\rho_{\rm min} \propto d^3_{\rm NV}/T^{3/2}_{\rm tot}$. If the molecular diffusion is fast compared to the NV T_2, then we choose $T_{\rm tot} \approx \tau_C = 2d^2_{\rm NV}/D$ [1]. Substituting this expression in the previous one, we conclude that $\rho_{\rm min}$ is independent of $d_{\rm NV}$. Intuitively, this is because a deeper NV feels a weaker $B^2_{\rm RMS}$ but can compensate by querying it for longer before the nuclear field randomizes.

Technique	Ref.	Analyte	B_0 field	RF pulses for nuclei	Volume	Number of spins	Detection
Cryogenic probe	[11]	^{1}H in 2 mM of sucrose in D_2O	14 T	yes	30 µL	6×10^{14} (1 nmol)	inductive
Atomic magnetometer	[12]	^{1}H in water	0.7 T prepolarize 0 T detection	yes	1 µL	7×10^{16} (120 nmol)	noninductive
Microcoil	[13]	^{1}H in water	1 T	yes	81 nL	3×10^{16} (50 nmol)	inductive
Microslot	[14]	^{1}H in 215 mM of sucrose in D_2O	11.7 T	yes	10.6 nL	3×10^{14} (0.5 nmol)	inductive
GMR	[15]	^{1}H in water	0.3 T	yes	62 pL	1×10^{17} (170 nmol)	noninductive
AMR	[16]	^{1}H in water	17 T prepolarize 0 T detection	yes	1 pL	3×10^{14} (0.5 nmol)	noninductive
Single NV	[6]	^{1}H in PPMA	40 mT	no	<1 fL	N/A	noninductive

Supplementary Table 3. **Small-volume NMR sensitivity assessment.** Overview of NMR techniques for small volumes. Here we list the number of spins detectable with SNR = 3 after 1 s of averaging.

Comparing $\rho_{\min}$ specifications in Fig. 1

Supplementary Table **3** summarizes the NMR techniques indicated in Fig. 1a in the main text. Compared to NV NMR spectroscopy, the microcoil/microslot/cryo-probe techniques have finer frequency resolution and can distinguish ^{1}H at different nuclear sites within a molecule. The determination of $\rho_{\min}$ under these techniques often focuses on a single site within a molecule, such as the anomeric proton in sucrose, so that the analyte concentration is the same as the detected spin concentration. For current implementations of NV NMR, chemical shifts are indistinguishable, so all nuclei of a given species contribute equally to the signal. Thus, when determining $\rho_{\min}$ in our technique, we must compute the concentration of all spins of the target species within the analyte, not just the concentration of analyte molecules.

SUPPLEMENTARY NOTE 2
NANOGRATING DIAMOND CHIPS AND EXPERIMENTAL DETAILS

Stopping and Range of Ions in Matter calculations

NV center depth profiles were estimated using the Stopping and Range of Ions in Matter (SRIM) Monte-Carlo simulation [17]. The diamond chips were modeled as a pure ^{12}C layer with 3.5 g/cm^3 density and 37.5 eV atom displacement threshold energy [18]. The lattice damage threshold and surface damage threshold were set to 7.35 eV and 7.5 eV, respectively. Note that SRIM simulations do not take into account crystallographic effects such as ion channeling, and therefore could lead to an underestimation of the NV implantation depth [19], but are sufficiently accurate for our purposes.

Supplementary Table **4** shows the implantation conditions studied here. One flat and one nanostructured diamond were implanted with each set of parameters, for a total of 6 chips. Both diamonds were mounted on the same substrate (as shown in Fig. 2d inset of main text) and were implanted twice, once at $\theta = +4°$ and once at $\theta = -4°$, where θ is the angle of incidence with respect to the

Chip name	Implant energy	Implant dose (2×)	Effective dose (2×)	Typical depth
UNM15 (flat)	20 keV	2×10^{13} cm^{-2}	1.4×10^{12} cm^{-2}	5 nm
UNM9 (grating)	20 keV	2×10^{13} cm^{-2}	1.4×10^{12} cm^{-2}	5 nm
UNM12 (flat)	60 keV	5×10^{13} cm^{-2}	3.5×10^{12} cm^{-2}	10 nm
UNM11 (grating)	60 keV	5×10^{13} cm^{-2}	3.5×10^{12} cm^{-2}	10 nm
UNM16 (flat)	200 keV	8×10^{13} cm^{-2}	5.6×10^{12} cm^{-2}	20 nm
UNM10 (grating)	200 keV	8×10^{13} cm^{-2}	5.6×10^{12} cm^{-2}	20 nm

Supplementary Table 4. **Diamond chip implantation summary.** Implantation parameters and SRIM characteristics for diamond chips studied in this work. The doses listed in the third and fourth columns were delivered twice, once at $\theta = +4°$ and once at $\theta = -4°$.

substrate surface normal vector. The effective dose is the dose delivered to a single face of a flat diamond or to the sidewalls of a nanograting chip. It is calculated as the area dose (ions/cm^2) multiplied by $\sin\theta$. Doses were selected that produce a nitrogen density of $\sim 10^{18}$ cm^{-3} [Supplementary Fig. **2**(a)], which is in the range previously found optimal for NV-ensemble sensing applications [4, 20]. This dose still yielded fewer than 10^{21} vacancies/cm^3 [Supplementary Fig. **2**(b)], far below the graphitization threshold of $\sim 10^{22}$ vacancies/cm^3 [21].

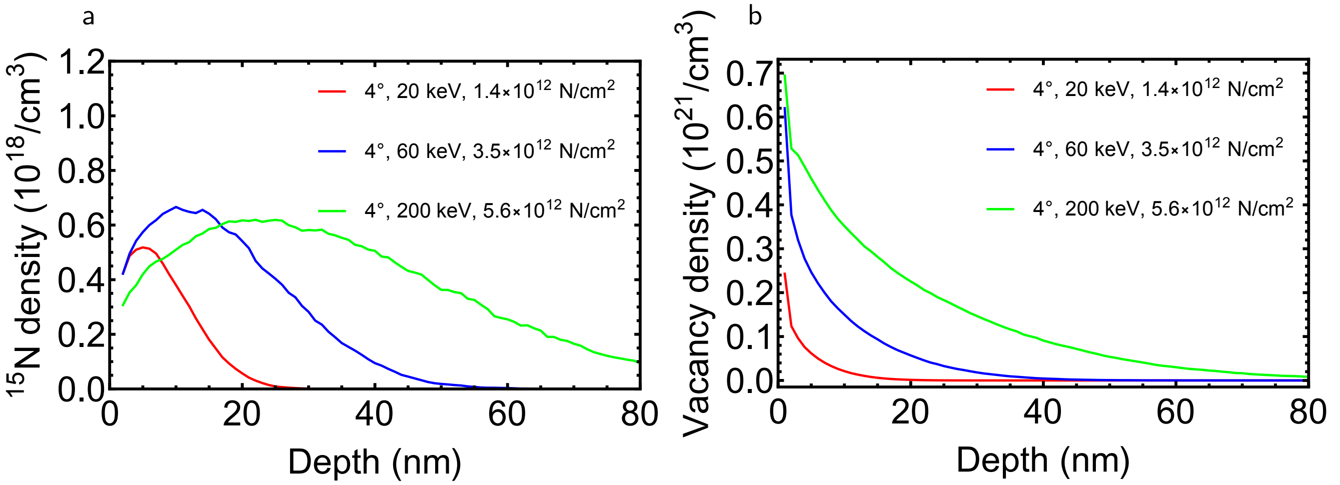

Supplementary Figure 2. **Implantation ^{15}N and vacancy densities.** SRIM ^{15}N and vacancy depth profiles for our implantation conditions (Supplementary Table **4**). Legends indicate the different implantation energies and effective doses (taking into account the factor of $\sin\theta$ described in the text).

Supplementary Fig. **2**(a) plots the nitrogen depth profile following implantation for different implantation conditions. Our SRIM simulations predict a ∼5 nm modal depth (the depth where nitrogen density is greatest) for 20 keV $^{15}N^+$ ion implantation, ∼10 nm depth for 60 keV, and ∼20 nm depth for 200 keV. The NV depth profiles should be similar to the simulated nitrogen profiles if there is relatively uniform nitrogen-to-NV conversion efficiency. To obtain similar NV density for all three implantation energies, we adjusted the nitrogen implantation doses to obtain approximately 0.5×10^{18} N/cm^{-3} = 3 ppm at the modal depth.

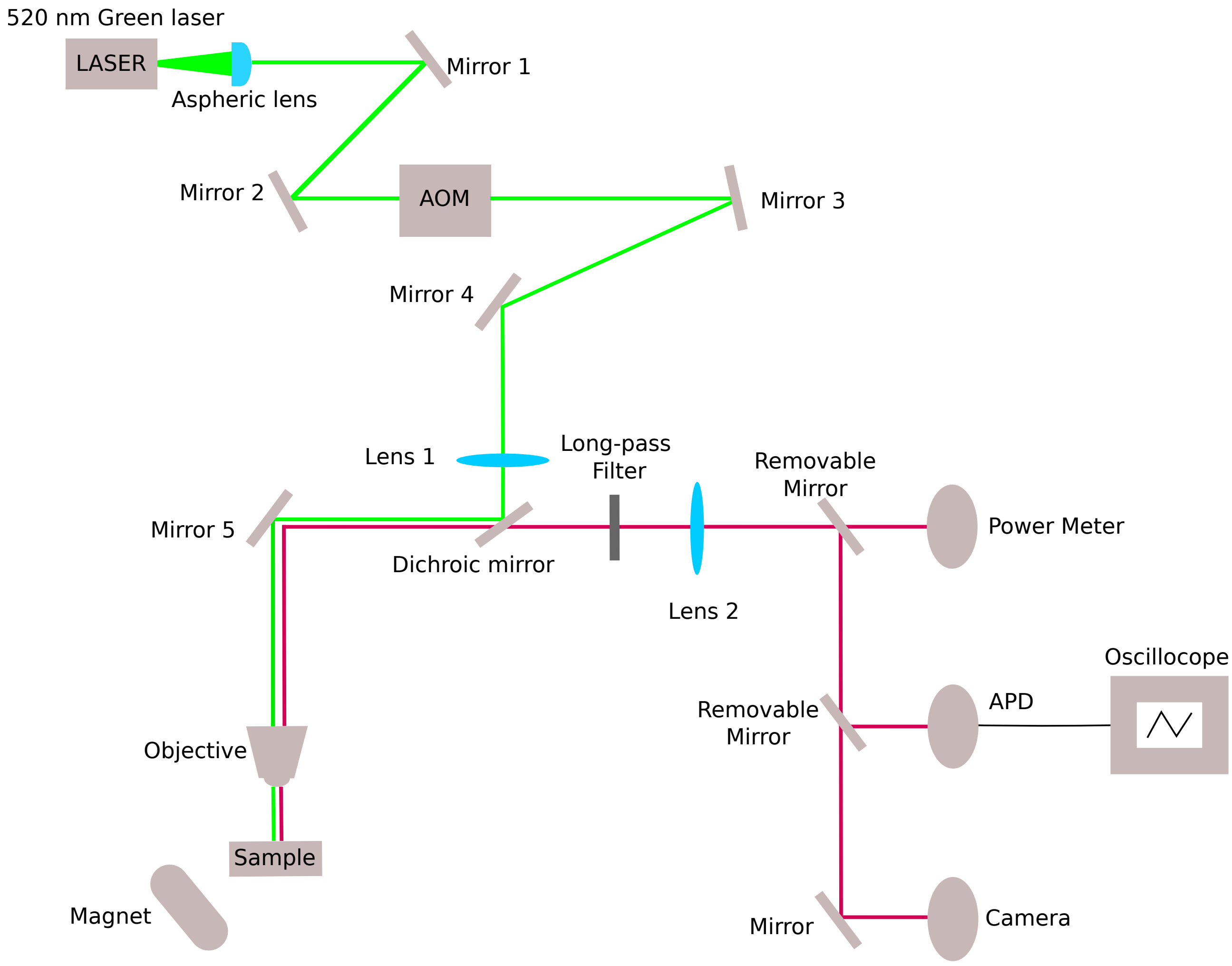

Supplementary Figure 3. **Experimental setup schematic.** A schematic of the epifluorescence microscope setup. The microwave source, amplifier, microwave switches, and acquisition computer are not shown. The computer controls the AOM and microwave switches with digital pulses and reads out averaged fluorescence time traces from the oscilloscope. Green lines indicate the 520 nm pump laser path and red lines indicate the NV fluorescence beam path.

Epifluorescence microscope setup

Supplementary Fig. 3 shows the experimental setup. Light from a 520 nm diode laser is used to pump and probe the NV centers (140 mW at the objective). The excitation beam is shaped to illuminate a $(25\ \mu\text{m})^2$ patch on the diamond. The oil-immersion microscope objective (1.25 NA, 100× magnification) collects the NV fluorescence, which is detected with an avalanche photodiode (APD). A camera is used to estimate the beam spot size and image the nanogratings, and a power meter to is used to monitor excitation power and fluorescence intensity. An oscilloscope measures the APD output voltage, reporting fluorescence time traces to the experimental control computer (not shown). The computer controls a I/Q modulated microwave generator, which is modulated with fast TTL pulses using microwave switches (not shown). The microwaves are amplified and applied to the diamond with a wire loop fabricated on a glass coverslip.

Diamond chip characterization

Chip name	Fluorescence	Rabi contrast	T_2 (176 pulses)	T_2 (1 pulse)	p exponent
UNM15 (flat)	4 nW	0.035	54±5 µs	N/A	N/A
UNM9 (grating)	76 nW	0.027	53±6 µs	4.1±0.5 µs	0.51±0.03
UNM12 (flat)	20 nW	0.035	83±5 µs	6.6±0.5 µs	0.49±0.02
UNM11 (grating)	580 nW	0.027	75±9 µs	3.2±0.5 µs	0.63±0.04
UNM16 (flat)	15 nW	0.019	73±8 µs	2.9±0.7 µs	0.63±0.06
UNM10 (grating)	795 nW	$\gtrsim$0.014	70±3 µs	2.7±0.5 µs	0.62±0.04

Supplementary Table 5. **Diamond chip characterization.** Chip characterization done with the epifluorescence microscope at $B_0 \approx 5$ mT aligned along one of the N-V axes. Here we list the fluorescence intensity when illuminated by 140 mW of 520 nm laser light collected through a 1.25 NA oil objective lens. The contrast represents the fluorescence difference of ($\sim$15 MHz) Rabi oscillations between the $m = 0$ and $m = -1$ states. The NV T_2 is evaluated for an XY8-22 pulse sequence (176 π-pulses). T_2 increases with more π-pulses with a power law dependence with exponent p.

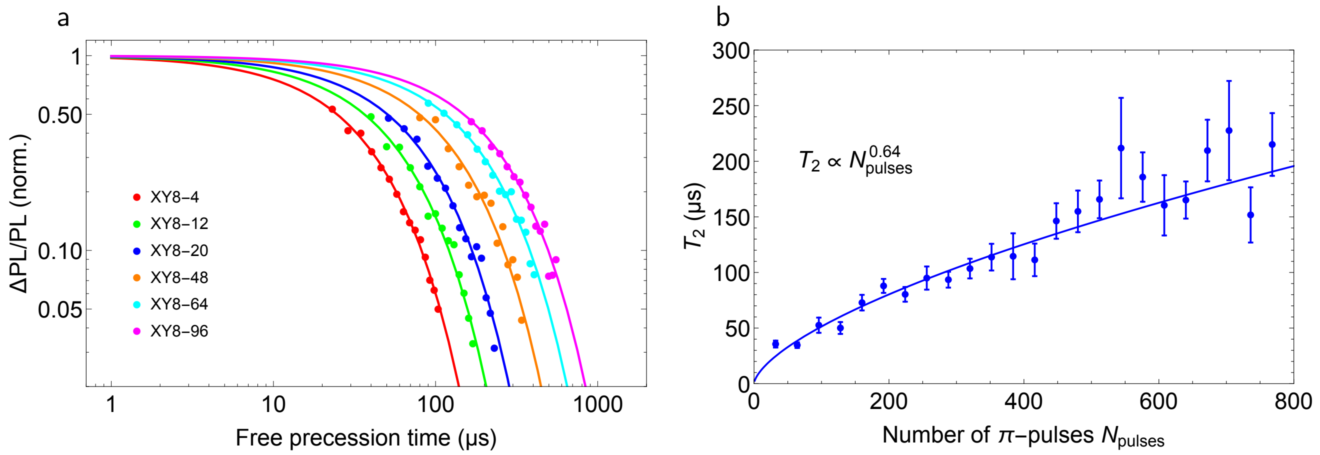

Supplementary Figure 4. **NV T_2 coherence lifetimes.** (a) XY8-N T_2 measurements for diamond chip UNM6 (a nanograting chip not included in the above tables; 33%:67% diamond/gap duty cycle, 40 keV implant, 5×10^{13} $^{15}N^+/cm^2$, ±5° degree implant angle). The plotted fit functions are exponential decays used to extract T_2. (b) Adding more π-pulses to the decoupling pulse sequence improves T_2 with a power law scaling. Error bars represent standard error of the mean (SEM).

As described in the main text, we demonstrate that the nanograting chips have brighter fluorescence without sacrificing NV coherence or contrast. Supplementary Table 5 lists the results of Fig. 2(d,e) in greater detail. Supplementary Fig. 4 shows the results of typical NV XY8-N experiments used to characterize the NV T_2 coherence. Note that the NMR experiments performed in this work required only a modest T_2 in the tens of µs range. This is because the maximum phase accumulation time is already restricted to this range due to rapid molecular diffusion within the analyte. As such, we were able to avoid using ^{12}C-enriched diamond layers and implant with a high $^{15}N^+$ density even though the magnetic noise from ^{13}C nuclei and paramagnetic nitrogen defects in the diamond tends to reduce the NV T_2 coherence time.

The nanograting chips can only exhibit improved NMR sensitivity if the analyte solution wets the diamond surface. NV centers from regions that do not wet are too far from analyte to register its

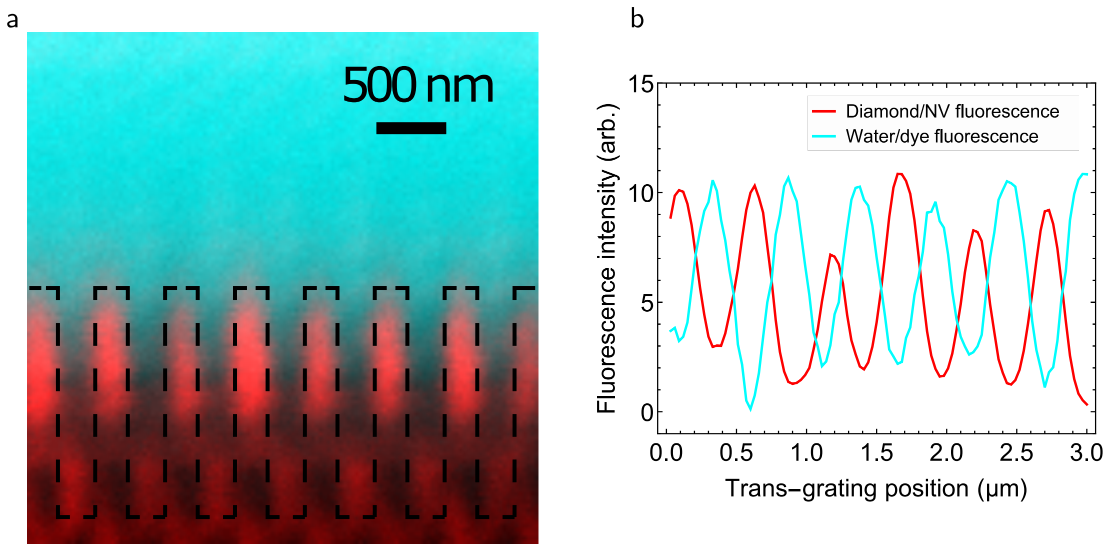

Supplementary Figure 5. Nanograting wetting. (a) Confocal microscopy image from Fig. 2(c) in the main text. (b) Line cut of fluorescence across the grating shows that fluorescence from water dye is anticorrelated with the fluorescence from diamond, confirming wetting. The confocal microscope point spread function is ∼0.3 μm in transverse directions and ∼1 μm in the longitudinal direction. This causes some dye fluorescence to appear to come from the diamond and vice versa.

NMR spectrum and thus reduce the overall signal contrast. Wetting can be a challenge with dense, high-aspect-ratio nanostructures, often depending on factors such as surface termination [22, 23]. To confirm that water wets the grating surfaces, we immersed a grating chip in an Alexa 405 dye/water solution and measured NV and dye fluorescence with a confocal microscope Leica TCS SP8 [Supplementary Fig. 5(a)]. Supplementary Fig. 5(b) shows that dye and NV fluorescence (when illuminated with blue and green light, respectively) are spatially anticorrelated. This confirms that the nanograting sidewalls are largely in contact with the analyte. The improved NMR sensitivity exhibited by nanograting chips reported in the manuscript is further confirmation, as this is also only possible if there is substantial wetting of the nanograting sidewalls.

Molecular diffusion analysis

To determine the effects of molecular diffusion, we acquired ^{1}H correlation data from all three nanograting chips with two different analytes, glycerol and Olympus Type-F microscope immersion oil. For the 200 keV chip (UNM10) with glycerol the correlation signal was too weak to properly analyze. We binned the NV correlation spectroscopy measurement values into different sets of ranges of $\tilde{\tau}$ and obtained the signal amplitude from each bin from its Fourier transform. We fit the signal amplitude versus time with a decaying exponential to extract the nuclear-spin correlation time τ_C (examples shown in Supplementary Fig. 6). We repeated using different binning lengths/techniques to obtain the approximate uncertainties σ_n for the error bars shown in Fig. 6(c) of the main text.

For fitting to a molecular diffusion model, we simulated 20, 40, 60, 80, and 200 keV implantation with the SRIM calculation described above. We extracted the modal implantation depth for each energy, and fit these data with a spline interpolation function to generate a smooth function, $f(E)$, that maps energy E to depth, $d_{\mathrm{NV}} = f(E) \times E$. We then fit the τ_C versus implantation energy data with the function $\tau_C = d_{\mathrm{NV}}^2/D = f(E)^2 \times E^2/D$, revealing the diffusion coefficient, D. We used a weighted fit (with the nth point in the fit weighted proportional to $1/\sigma_n^2$) because the 20

keV correlation times were extracted with small relative uncertainties while the 200 keV correlation measurements were noisier.

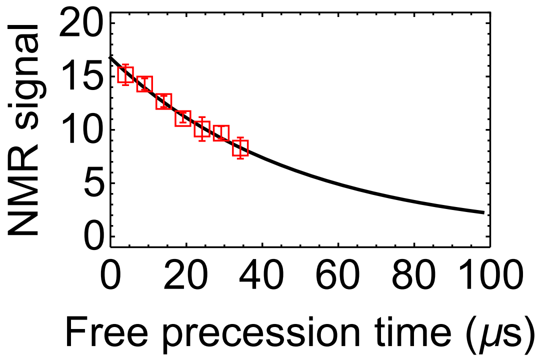

Supplementary Figure 6. **Molecular diffusion correlation decay (glycerol).** A glycerol molecular diffusion NMR decay curve measured using the technique described in this text. The sensor was UNM11 (60 keV grating chip). Error bars represent standard error of the mean (SEM).

Narrowest-observed spectral resolution

The NV T_1 time sets the best-case correlation spectroscopy linewidth to $(\pi T_1)^{-1} \approx 100$ Hz full-width-at-half-maximum (FWHM), which is often unachievable due to molecular diffusion broadening. Supplementary Fig. 7 shows the narrowest NMR peak, 3.5 kHz FWHM, we observed using a 40 keV implant nanograting chip (not included in the main analysis presented). This especially narrow linewidth may be due to the "hardened oil effect" causing reduced molecular diffusion near the diamond surface [24], and emphasizes that restricting diffusion is important to fully exploit correlation NMR spectroscopy with shallow NVs.

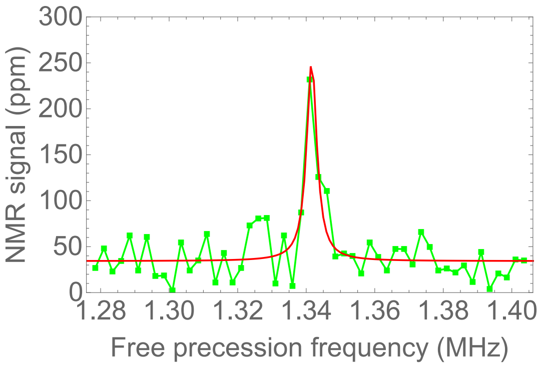

Supplementary Figure 7. **A 3.5 kHz proton linewidth.** A ^{1}H NMR spectrum for a correlation spectroscopy measurement with nanograting chip UNM6 (not listed in the above tables; 33%:67% diamond/gap duty cycle, 40 keV implant, 5×10^{13} $^{15}N^+/cm^2$, $\pm 5°$ degree implant angle) with microscope immersion oil ($B_0 = 31.5$ mT). This is the narrowest NMR line we measured in our experiments, with a full-width-at-half-maximum (FWHM) of 3.5 kHz.

SUPPLEMENTARY REFERENCES

[1] Linh M. Pham, Stephen J. DeVience, Francesco Casola, Igor Lovchinsky, Alexander O. Sushkov, Eric Bersin, Junghyun Lee, Elana Urbach, Paola Cappellaro, Hongkun Park, Amir Yacoby, Mikhail Lukin, and Ronald L. Walsworth, "NMR technique for determining the depth of shallow nitrogen-vacancy centers in diamond," Phys. Rev. B **93**, 045425 (2016).

[2] B. E. Herzog, D. Cadeddu, F. Xue, P. Peddibhotla, and M. Poggio, "Boundary between the thermal and statistical polarization regimes in a nuclear spin ensemble," Applied Physics Letters **105**, 043112 (2014).

[3] Abdelghani Laraoui, Florian Dolde, Christian Burk, Friedemann Reinhard, Jörg Wrachtrup, and Carlos A. Meriles, "High-resolution correlation spectroscopy of ^{13}C spins near a nitrogen-vacancy centre in diamond," Nature Communications **4**, 1651 (2013).

[4] V. M. Acosta, E. Bauch, M. P. Ledbetter, C. Santori, K.-M. C. Fu, P. E. Barclay, R. G. Beausoleil, H. Linget, J. F. Roch, F. Treussart, S. Chemerisov, W. Gawlik, and D. Budker, "Diamonds with a high density of nitrogen-vacancy centers for magnetometry applications," Phys. Rev. B **80**, 115202 (2009).

[5] D. Farfurnik, A. Jarmola, L. M. Pham, Z. H. Wang, V. V. Dobrovitski, R. L. Walsworth, D. Budker, and N. Bar-Gill, "Optimizing a dynamical decoupling protocol for solid-state electronic spin ensembles in diamond," Phys. Rev. B **92**, 060301 (2015).

[6] D. Rugar, H. J. Mamin, M. H. Sherwood, M. Kim, C. T. Rettner, K. Ohno, and D. D. Awschalom, "Proton magnetic resonance imaging using a nitrogen-vacancy spin sensor," Nature Nanotechnology **10**, 120–124 (2015).

[7] M. Loretz, S. Pezzagna, J. Meijer, and C. L. Degen, "Nanoscale nuclear magnetic resonance with a 1.9-nm-deep nitrogen-vacancy sensor," Applied Physics Letters **104**, 033102 (2014).

[8] Mary W. Fey, C. M. Weil, and J. B. Segur, "Solubility of Sucrose in Aqueous Glycerol and Propylene Glycol," Industrial & Engineering Chemistry **43**, 1435–1436 (1951).

[9] John Burgess, Metal ions in solution (Halsted Press, 1978).

[10] S. R. Gregory, "Physical Properties of Glycerine," in Glycerine: A Key Cosmetic Ingredient, edited by Eric Jungermann and Norman O.V. Sonntag (CRC Press, 1991) Chap. 6, pp. 113–156.

[11] Helena Kovacs, Detlef Moskau, and Manfred Spraul, "Cryogenically cooled probes-a leap in NMR technology," Progress in Nuclear Magnetic Resonance Spectroscopy **46**, 131 – 155 (2005).

[12] M. P. Ledbetter, I. M. Savukov, D. Budker, V. Shah, S. Knappe, J. Kitching, D. J. Michalak, S. Xu, and A. Pines, "Zero-field remote detection of NMR with a microfabricated atomic magnetometer," Proceedings of the National Academy of Sciences **105**, 2286–2290 (2008).

[13] Andrew F. McDowell and Natalie L. Adolphi, "Operating nanoliter scale NMR microcoils in a 1 tesla field," Journal of Magnetic Resonance **188**, 74 – 82 (2007).

[14] Hans Georg Krojanski, Jörg Lambert, Yilmaz Gerikalan, Dieter Suter, and Roland Hergenroder, "Microslot NMR Probe for Metabolomics Studies," Analytical Chemistry **80**, 8668–8672 (2008).

[15] P. A. Guitard, R. Ayde, G. Jasmin-Lebras, L. Caruso, M. Pannetier-Lecoeur, and C. Fermon, "Local nuclear magnetic resonance spectroscopy with giant magnetic resistance-based sensors," Applied Physics Letters **108**, 212405 (2016).

[16] F. Verpillat, M. P. Ledbetter, S. Xu, D. J. Michalak, C. Hilty, L.-S. Bouchard, S. Antonijevic, D. Budker, and A. Pines, "Remote detection of nuclear magnetic resonance with an anisotropic magnetoresistive sensor," Proceedings of the National Academy of Sciences **105**, 2271–2273 (2008).

[17] James F. Ziegler, M.D. Ziegler, and J.P. Biersack, "SRIM - the stopping and range of ions in matter (2010)," Nuclear Instruments and Methods in Physics Research Section B: Beam Interactions with Materials and Atoms **268**, 1818 – 1823 (2010), 19th International Conference on Ion Beam Analysis.

[18] J. Koike, D. M. Parkin, and T. E. Mitchell, "Displacement threshold energy for type iia diamond," Applied Physics Letters **60**, 1450–1452 (1992).

[19] David M. Toyli, Christoph D. Weis, Gregory D. Fuchs, Thomas Schenkel, and David D. Awschalom, "Chip-Scale Nanofabrication of Single Spins and Spin Arrays in Diamond," Nano Letters **10**, 3168–3172 (2010).

[20] Stephen J. DeVience, Linh M. Pham, Igor Lovchinsky, Alexander O. Sushkov, Nir Bar-Gill, Chinmay Belthangady, Francesco Casola, Madeleine Corbett, Huiliang Zhang, Mikhail Lukin, Hongkun Park, Amir Yacoby, and Ronald L. Walsworth, "Nanoscale NMR spectroscopy and imaging of multiple nuclear species," Nature Nanotechnology **10**, 129–134 (2015).

[21] C. Uzan-Saguy, C. Cytermann, R. Brener, V. Richter, M. Shaanan, and R. Kalish, "Damage threshold for ion-beam induced graphitization of diamond," Applied Physics Letters **67**, 1194–1196 (1995).

[22] Deying Xia and S. R. J. Brueck, "Strongly Anisotropic Wetting on One-Dimensional Nanopatterned Surfaces," Nano Letters **8**, 2819–2824 (2008).

[23] Deying Xia, Xiang He, Ying-Bing Jiang, Gabriel P. Lopez, and S. R. J. Brueck, "Tailoring Anisotropic Wetting Properties on Submicrometer-Scale Periodic Grooved Surfaces," Langmuir **26**, 2700–2706 (2010).

[24] T. Staudacher, N. Raatz, S. Pezzagna, J. Meijer, F. Reinhard, C. A. Meriles, and J. Wrachtrup, "Probing molecular dynamics at the nanoscale via an individual paramagnetic centre," Nature Communications **6**, 8527 (2015).
